# 

**Published:** 1887-06

**Authors:** 


### President.

F. POOLE LANSDOWN.

### President-Elect.

L. M. GRIFFITHS.

### Honorary Secretary.

R. SHINGLETON SMITH, M.D., B.Sc. Lond.

### Committee.

*Past Presidents.*
- R. W. COE.
- AUGUSTIN PRICHARD, M.D. Berlin.
- J. G. SWAYNE, M.D. Lond.
- E. LONG FOX, M.D. Oxon.
- JAMES G. DAVEY, M.D. St. And.
- W. JOHNSTONE FYFFE, M.D. Dub.
- G. F. BURDER, M.D. Aberd.
- W. H. SPENCER, M.D., M.A. Cantab.

F. RICHARDSON CROSS, M.B. Lond.
NELSON C. DOBSON.
A. J. HARRISON, M.B. Lond.
W. H. HARSANT.
E. MARKHAM SKERRITT, M.D., B.S., B.A. Lond.
J. GREIG SMITH, M.B., C.M., M.A. Aberd., F.R.S.E.

Medical Men wishing to join the Society are requested to communicate with the Honorary Secretary, Dr. R. SHINGLETON SMITH, 9 Richmond Hill, Clifton. The Annual Subscription is 10/6.

---

*Extracts from Journal By-laws.*

4.—The *Journal* shall be delivered free to Subscribers and also to Members of the Society whose subscriptions are not in arrear.

6.—The Annual Subscription to the *Journal* for those who pay before the 1st of July shall be 5/-, post free, or 6/- if paid after that date.

---

Communications intended for insertion in the *Journal* should be sent to the Editor, J. GREIG SMITH, M.A., F.R.S.E., 16 Victoria Square, Clifton.

Books for Review, Exchange Journals, and communications referring to the delivery of the *Journal* should be sent to the Assistant-Editor, L. M. GRIFFITHS, 9 Gordon Road, Clifton, to whom Subscriptions are to be paid *in advance*.

Authors requiring Reprints of their Papers should communicate with the Publisher.

Cases for Binding Vols. I., II., III., and IV. are now ready, and may be obtained, price 7*d.* each (postage 2*d.* extra), upon application to J. W. ARROWSMITH, Quay Street, Bristol; or the *Journal* will be bound, including Case, for 1/3 per volume.

### ABSTRACT REPORT.

This Society was founded on a strictly and wholly mutual basis, at a meeting of members of the medical profession, summoned by Mr. Earnest Hart, at the time of the Annual Meeting of the British Medical Association in August, 1883, and commenced its opperations in March, 1884. Its purpose is to enable members of the Medical profession to provide, by limited annual payments, against sickness, accident and disablement from practice (temporary or permanent), and also for Life Assurance. It has accumulated during three years and a half a Reserve Fund amounting to £17,645, has now an Annual Premium Revenue of over £8,000, and has during the same period paid £3,878 for sickness to members in every part of the United Kingdom, and £500 on account of deaths.

There are no payments for management expenses, except the cost of secretarial and official work, no outlay for agencies, or commissions.

The economy of management is such that, although in the first instance, the unusually small proportion of ten per cent. of the premium income was allowed for this purpose, the actual expenditure has been less than five per cent., and a fund of £1,448 has already been accumulated on this account, and stands as a profit saved for and on behalf of the members.

The amount added to the reserves during the year ending June 30, 1887, was £6,314, and the accumulated funds of the Society stand invested in the names of the Trustees in high-class securities for the sole advantage of the members.

There are no shares or shareholders, and the whole of the surpluses will accrue to the benefit of the members.

Owing to the mutual character of the Society, and to the absence of the usual fees and charges for management, the rates of payment are below those of any other comparable organization. The tables have been calculated by an eminent actuary (Mr. F. G. P. Neison, F.R.I.A.), and the experience of the Society has shown that in each department the fullest allowance has been made for contingencies.

No accession to numbers is needed to ensure the continued stability and profitable working of the Society, but it is desired to extend the benefits which it offers for assuring against sickness, accident, and disablement to all members of the profession.

As the Society is of an entirely mutual and independent character, and in no sense eleemosynary, its members are able to fulfil the duty of self-help, and avail themselves of the provision made for themselves by themselves without hesitation or any sacrifice of dignity or self-respect.

Full particulars, Proposal Forms, Reports, and other documents will be supplied by Mr. C. J. Radley, Secretary, 26, Wynne Road, Brixton, S.W.

*Complete in Three Volumes, each containing about* 600 *pages fcap.* 8*vo, fully Illustrated.* **7s. 6d.** *each.*

### A Manual of Surgery.

In Treatises by various Authors. Edited by FREDERICK TREVES, F.R.C.S., Surgeon to, and Lecturer on Anatomy at, the London Hospital, Hunterian Professor at the Royal College of Surgeons of England; and containing contributions by leading Physicians and Surgeons. The work forms a complete, concise, and authoritative Treatise on the Science and Art of Modern Surgery.

*Cassell & Company, Limited, Ludgate Hill, London.*

---

384 *pages, demy* 8*vo, with* 6 PLATES. *Price* **21s.**

### Memorials of the Craft of Surgery in England.

From materials compiled by JOHN FLINT SOUTH, twice President of the Royal College of Surgeons of England, and Surgeon to St. Thomas's Hospital. Edited by D'ARCY POWER, M.A. Oxon., F.R.C.S. Eng. With an Introduction by Sir JAMES PAGET.

*Cassell & Company, Limited, Ludgate Hill, London.*

---

*Price* **One Guinea** *per annum, post free, or* **6s.** *per part.*

### The International Journal of the Medical Sciences.

Edited by I. MINIS HAYS, M.D., Philadelphia, and MALCOLM MORRIS, London.

*Issued quarterly—in January, April, July, and October.*

Its main features are :—

1. **Original Articles and Monographs.**
2. **Elaborate Reviews of all the chief Contributions to Medical Literature.**
3. **Quarterly Summaries of the Progress made in all Departments of Medicine, general as well as special.**

The contributors to the Journal include the leading Physicians, Surgeons, and Scientists in Great Britain and America.

*Cassell & Company, Limited, Ludgate Hill, London.*

### MANUALS

FOR

### Students of Medicine.

THIS Series has been projected to meet the demand of Medical Students and Practitioners for compact and authoritative Manuals embodying the most recent discoveries, and presenting them to the reader in a cheaper and more portable form than has till now been customary in Medical Works.

The Manuals contain all the information required for the Medical Examinations of the various Colleges, Halls, and Universities in the United Kingdom and the Colonies.

The Authors will be found to be either Examiners or the leading Teachers in well-known Medical Schools. This ensures the practical utility of the Series, while the introduction of the results of the latest scientific researches, British and Foreign, will recommend them also to Practitioners who desire to keep pace with the swift strides that are being made in Medicine and Surgery.

In the rapid advance in modern Medical knowledge, new subjects have come to the front which have not as yet been systematically handled, nor the facts connected with them properly collected. The treatment of such subjects forms an important feature of this Series.

New and valuable Illustrations are freely introduced. The Manuals are printed in clear type, upon good paper. They are of a size convenient for the pocket, and bound in red cloth limp, with red edges. They contain from 300 to 540 pages, and are published at prices varying from 5s. to 7s. 6d.

### Manuals for Students of Medicine.

*Published by CASSELL & COMPANY.*

---

**Elements of Histology.** By E. KLEIN, M.D., F.R.S., Joint-Lecturer on General Anatomy and Physiology in the Medical School of St. Bartholomew's Hospital, London. *Fifth Edition.* 6s.

**Surgical Pathology.** By A. J. PEPPER, M.B., M.S., F.R.C.S., Surgeon and Teacher of Practical Surgery at St. Mary's Hospital. *Second Edition.* 7s. 6d.

**Surgical Applied Anatomy.** By FREDERICK TREVES, F.R.C.S., Surgeon to, and Lecturer on Anatomy at, the London Hospital. *Second Edition.* 7s. 6d.

**Clinical Chemistry.** By CHARLES H. RALFE, M.D., F.R.C.P., Assistant Physician at the London Hospital. 5s.

**Human Physiology.** By HENRY POWER, M.B., F.R.C.S., Examiner in Physiology, Royal College of Surgeons of England. *Second and Enlarged Edition.* 7s. 6d.

**Materia Medica and Therapeutics.** By J. MITCHELL BRUCE, M.D., F.R.C.P., Lecturer on Materia Medica at Charing Cross Medical School, and Physician to the Hospital. *Fourth Edition.* Containing an account of the action and uses of all the important new Drugs admitted into the Pharmacopœia. 7s. 6d.

**Physiological Physics.** By J. MCGREGOR-ROBERTSON, M.A., M.B., Muirhead Demonstrator of Physiology, University of Glasgow. 7s. 6d.

**Surgical Diagnosis: A Manual for the Wards.** By A. PEARCE GOULD, M.S., M.B., F.R.C.S., Assistant Surgeon to Middlesex Hospital. 7s. 6d.

**Comparative Anatomy and Physiology.** By F. JEFFREY BELL, M.A., Professor of Comparative Anatomy at King's College. 7s. 6d.

**Forensic Medicine.** By A. J. PEPPER, M.S., M.B., F.R.C.S., Examiner in Forensic Medicine to the University of London.

**A Manual of Surgery.** Edited by FREDERICK TREVES, F.R.C.S. With contributions by leading Physicians and Surgeons. Complete in Three Volumes, each containing about 600 pages fcap. 8vo, fully Illustrated. 7s. 6d. each.

*Other Volumes will follow in due course.*

*Cassell & Company, Limited, Ludgate Hill, London.*

### Clinical Manuals

FOR

*Practitioners and Students of Medicine.*

**Complete Monographs on Special Subjects.**

---

"A valuable series, which is likely to form, when completed, perhaps the most important Encyclopædia of Medicine and Surgery in the English language."—*British Medical Journal.*

---

THE object of this Series is to present to the Practitioner and Student of Medicine original, concise, and complete monographs on all the principal subjects of Medicine and Surgery, both general and special.

It is hoped that the Series will enable the Practitioner to keep abreast with the rapid advances at present being made in medical knowledge, and that it will supplement for the Student the comparatively scanty information on special subjects contained in the general text-books.

The Series will form a complete Encyclopædia of Medical and Surgical Science in separate volumes.

The Manuals are written by leading Hospital Physicians and Surgeons, whose work on each special subject may be considered to be authoritative.

The Manuals are printed in clear type upon good paper. They are of a size convenient for the pocket, substantially bound in cloth limp. Each volume contains about 544 pages, and is freely Illustrated, when required, by Original Chromo-Lithographs and Woodcuts.

*LIST OF MANUALS.*

***Fractures and Dislocations.*** By T. PICKERING PICK, F.R.C.S., Surgeon to, and Lecturer on Surgery at St. George's Hospital. **8s. 6d.**

***Surgical Diseases of the Kidney.*** By HENRY MORRIS, M.B., F.R.C.S., Surgeon to, and Lecturer on Surgery at, Middlesex Hospital. With 6 chromo plates, **9s.**

***Insanity and Allied Neuroses.*** By GEORGE H. SAVAGE, M.D., Medical Superintendent and Resident Physician to Bethlem Royal Hospital, and Lecturer on Mental Diseases at Guy's Hospital. **8s. 6d.**

***Intestinal Obstruction.*** By FREDERICK TREVES, F.R.C.S., Surgeon to, and Lecturer on Anatomy at, the London Hospital. **8s. 6d.**

***Diseases of the Tongue.*** By H. T. BUTLIN, F.R.C.S., Assistant Surgeon to St. Bartholomew's Hospital. With 8 chromo plates. **9s.**

***Surgical Diseases of Children.*** By EDMUND OWEN, M.B., F.R.C.S., Surgeon to the Children's Hospital, Great Ormond Street, and Surgeon to, and Lecturer on Anatomy at, St. Mary's Hospital. With 4 chromo plates. **9s.**

***Syphilis.*** By JONATHAN HUTCHINSON, F.R.S., F.R.C.S., Consulting Surgeon to the London Hospital and to the Royal London Ophthalmic Hospital. With 8 chromo plates, **9s.**

***Diseases of Joints.*** By HOWARD MARSH, F.R.C.S., Senior Assistant Surgeon to, and Lecturer on Anatomy at, St. Bartholomew's Hospital, and Surgeon to the Children's Hospital, Great Ormond Street. With Chromo Frontispiece, **9s.**

***Ophthalmic Surgery.*** By R. BRUDENELL CARTER, F.R.C.S., Ophthalmic Surgeon to, and Lecturer on Ophthalmic Surgery at St. George's Hospital; and W. ADAMS FROST, F.R.C.S., Assistant Ophthalmic Surgeon to, and Joint Lecturer on Ophthalmic Surgery at St. George's Hospital.

***The Pulse.*** By W. H. BROADBENT, M.D., F.R.C.P., Physician to, and Lecturer on Medicine at, St. Mary's Hospital.

***Diseases of the Breast.*** By THOMAS BRYANT, F.R.C.S., Surgeon to, and Lecturer on Surgery at, Guy's Hospital.

***Diseases of the Rectum and Anus.*** By CHARLES B. BALL, M.Ch. (Dublin), F.R.C.S.I., Surgeon and Clinical Teacher at Sir P. Dun's Hospital.

*Other Volumes will follow in due course.*

*Cassell & Company, Limited, Ludgate Hill, London.*

*Issued Yearly.* Price 5s.

### THE YEAR-BOOK OF TREATMENT.

*A Critical Review for Practitioners of Medicine.*

THE object of this book is to present to the Practitioner not only a complete account of all the more important advances made in the Treatment of Disease, but to furnish also a Review of the same by a competent authority.

Each department of practice is fully and concisely treated, and such allusions to recent pathological and clinical work as bear directly upon Treatment enter into the consideration of each subject.

The medical literature of all countries is placed under contribution, and the Work deals with all matters relating to Treatment that have been published during the year ending Sept. 30th. *A full reference is given to every article noticed.*

---

"This book is the combined work of twenty three contributors, who have not only abstracted the best contributions to the practice of medicine and surgery during the twelve months, but have criticised them. The whole is compressed into 300 octavo pages, and the matter may be said to lie in a nutshell. The work appears to have been apportioned to the individual contributors with excellent judgment, and the result is *a book of extreme value* to all who in these busy times find it difficult to keep pace with the ever-advancing march of the science and art of medicine."—*Lancet.*

"This handbook contains, within the space of three hundred pages, a wonderfully complete summary—review of the methods of treatment, new and resuscitated, which have been advocated during the year with which it deals."—*British Medical Journal.*

"*We can strongly recommend the book to all practitioners,* and we shall not be exaggerating if we say that this book, which we are pleased to see has already reached a second edition, is one of that class which it is an economy to possess."—*London Medical Record.*

*Cassell & Company, Limited, Ludgate Hill, London.*

*Authoritative Work on Health by Eminent Physicians and Surgeons.*

### The Book of Health.

A Systematic Treatise for the Professional and General Reader upon the Science and the Preservation of Health . ***21s.***
Half morocco . . . . . . . ***25s.***

CONTENTS.

By W. S. SAVORY, F.R.S.—INTRODUCTORY.
By SIR RISDON BENNETT, M.D., F.R.S.—FOOD AND ITS USE IN HEALTH.
By T. LAUDER BRUNTON, M.D., F.R.S.—THE INFLUENCE OF STIMULANTS AND NARCOTICS ON HEALTH.
By SIR J. CRICHTON-BROWNE, LL.D., M.D.—EDUCATION AND THE NERVOUS SYSTEM.
By JAMES CANTLIE, F.R.C.S.—THE INFLUENCE OF EXERCISE ON HEALTH.
By FREDERICK TREVES, F.R.C.S.—THE INFLUENCE OF DRESS ON HEALTH.
By J. E. POLLOCK, M.D.—THE INFLUENCE OF OUR SURROUNDINGS ON HEALTH.
By J. RUSSELL REYNOLDS, M.D., F.R.S.—THE INFLUENCE OF TRAVELLING ON HEALTH.
By SHIRLEY MURPHY, M.R.C.S.—HEALTH AT HOME.
By W. B. CHEADLE, M.D.—HEALTH IN INFANCY AND CHILDHOOD.
By CLEMENT DUKES, M.D.—HEALTH AT SCHOOL.
By HENRY POWER, F.R.C.S.—THE EYE AND SIGHT.
By G. P. FIELD, M.R.C.S.—THE EAR AND HEARING.
By J. S. BRISTOWE, M.D., F.R.S.—THE THROAT AND VOICE.
By CHARLES S. TOMES, F.R.S.—THE TEETH.
By MALCOLM MORRIS.—THE SKIN AND HAIR.
By SIR JOSEPH FAYRER, K.C.S.I., F.R.S., and J. EWART, M.D.—HEALTH IN INDIA.
By HERMANN WEBER, M.D.—CLIMATE AND HEALTH RESORTS.

Edited by MALCOLM MORRIS.

"A volume which deserves high praise throughout, and which will find its uses in every household."—*Times.*

"The work is what it aims to be—authoritative—and must become a standard work of reference not only with those who are responsible for the health of schools, workshops, and other establishments where there is a large concourse of individuals, but to every member of the community who is anxious to secure the highest possible degree of healthy living for himself and for his family."—*Lancet.*

---

HEALTH HANDBOOKS.

### The Influence of Clothing on Health.

By FREDERICK TREVES, F.R.C.S., Surgeon to, and Lecturer on Anatomy at, the London Hospital. 2s.

### The Eye, Ear, and Throat (The Management of).

The Eye and Sight. By HENRY POWER, M.B., F.R.C.S. The Ear and Hearing. By GEORGE P. FIELD. The Throat, Voice, and Speech. By JOHN S. BRISTOWE, M.D., F.R.S. 3s. 6d.

### The Skin and Hair (The Management of).

By MALCOLM MORRIS, F.R.C.S. Ed. 2s.

### Health at School.

By CLEMENT DUKES, M.D., B.S., Physician to Rugby School and to Rugby Hospital. 7s. 6d.

*Cassell & Company, Limited, Ludgate Hill, London.*

"An Encyclopædia of Sanitation."—SPECTATOR.

### *Our Homes, and How to Make them Healthy.*

With numerous Practical Illustrations. Edited by SHIRLEY FORSTER MURPHY, *late Medical Officer of Health to the Parish of St. Pancras; Hon. Secretary to the Epidemiological Society, and to the Society of Medical Officers of Heal'h.* 960 pages. Royal 8vo, cloth . . . . ***15s.***
Half morocco . . . . . ***21s.***

CONTENTS.

**Health in the Home.** By W. B. RICHARDSON, M.D., LL.D., F.R.S.

**Architecture.** By P. GORDON SMITH, F.R.I.B.A., and KEITH DOWNES YOUNG, A.R.I.B.A.

**Internal Decoration.** By ROBERT W. EDIS, F.S.A., and MALCOLM MORRIS, F.R.C.S. Ed.

**Lighting.** By R. BRUDENELL CARTER, F.R.C.S.

**Warming and Ventilation.** By DOUGLAS GALTON, C.B., D.C.L., F.R.S.

**House Drainage.** By WILLIAM EASSIE, C.E., F.L.S., F.G.S.

**Defective Sanitary Appliances and Arrangements.** By PROF. W. H. CORFIELD, M.A., M.D.

**Water.** By PROF. F. S. B. FRANÇOIS DE CHAUMONT, M.D., F.R.S.; ROGERS FIELD, B.A., M.I.C.E.; and J. WALLACE PEGGS, C.E.

**Disposal of Refuse by Dry Methods.** By THE EDITOR.

**The Nursery.** By WILLIAM SQUIRE, M.D., F.R.C.P.

**House Cleaning.** By PHYLLIS BROWNE.

**Sickness in the House.** By THE EDITOR.

**Legal Responsibilities.** By THOS. ECCLESTON GIBB.

&c. &c.

"A large amount of useful information concerning all the rights, duties, and privileges of a householder, as well as about the best means of rendering the home picturesque, comfortable, and, above all, wholesome."—*Times.*

---

*Fifth and Cheap Edition.* Price 1s. 6d.; cloth, 2s.

### *A Handbook of Nursing*

For the Home and for the Hospital. By CATHERINE J. WOOD, *Lady Superintendent of the Hospital for Sick Children, Great Ormond Street.*

---

CASSELL & COMPANY'S COMPLETE CATALOGUE, *containing particulars of several Hundred Volumes, including Bibles and Religious Works, Illustrated and Fine-Art Volumes, Children's Books, Dictionaries, Educational Works, History, Natural History, Household and Domestic Treatises, Science, Travels, &c., together with a Synopsis of their numerous Illustrated Serial Publications, sent post free on application.*

CASSELL & COMPANY, LIMITED, *Ludgate Hill, London. Paris, New York, & Melbourne.*

